# T cell receptor affinity and avidity defines antitumor response and autoimmunity in T cell immunotherapy

**DOI:** 10.1186/2051-1426-1-S1-P242

**Published:** 2013-11-07

**Authors:** Michelle Krogsgaard, Shi Zhong, Karolina Malecek, Laura A Johnson, Zhiya Yu, Eleazar Vega-Saenz de Miera, Farbod Darvishian, Katelyn McGary-Shipper, Kevin Huang, Joshua Boyer, Emily Corse, Yongzhao Shao, Steven A Rosenberg, Nicholas P Restifo, Iman Osman

**Affiliations:** 1 NYU Cancer Institute, New York University School of Medicine, New York, NY, USA; 2 Center for Cancer Research, National Cancer Institute, National Cancer Institute, NIH, Bethesda, MD, USA; 3 Department of Pathology, New York University School of Medicine, New York, NY, USA; 4 Department of Immunology, The University of Texas MD Anderson Cancer Center, Houston, TX, USA; 5 Abramson Family Cancer Research Institute, Perelman School of Medicine, University of Pennsylvania, Philadelphia, PA, USA; 6 Program in Structural Biology, New York University School of Medicine, New York, NY, USA; 7 Interdisciplinary Melanoma Cooperative Group, New York University School of Medicine, New York, NY, USA; 8 Division of Biostatistics, New York University School of Medicine, New York, NY, USA; 9 Ronald O. Perelman Department of Dermatology, New York University School of Medicine, New York, NY, USA

## 

T-cells have evolved the unique ability to discriminate "self" from "non-self" with high sensitivity and selectivity. However, tissue-specific autoimmunity, tolerance or eradication of cancer does not fit into the self/non-self paradigm because the T-cell responses in these situations are most often directed to non-mutated self-proteins. To determine the TCR affinity threshold defining the optimal balance between effective antitumor activity and autoimmunity in vivo, we used a novel self-antigen system comprised of seven human melanoma gp100_209-217_-specific TCRs spanning physiological affinities (1 to 100 μM). We found that in vitro and in vivo T cell responses are determined by TCR affinity. Strikingly, we found that T cell antitumor activity and autoimmunity are closely coupled but plateau at a defined TCR affinity of 10 µM, likely due to diminished contribution of TCR affinity to avidity above the threshold. Our results suggest a relatively low affinity threshold is necessary for the immune system to avoid self-damage given the close relationship between antitumor activity and autoimmunity. This, in turn, indicates that treatment strategies focusing on TCRs in the intermediate affinity range (KD ~10 μM) or targeting or targeting shared tumor antigens would dampen the potential for autoimmunity during adoptive T cell therapy for the treatment of cancer.

